# Scanning Near-Field Optical Microscopy of Ultrathin Gold Films

**DOI:** 10.3390/nano13081376

**Published:** 2023-04-15

**Authors:** Dmitry I. Yakubovsky, Dmitry V. Grudinin, Georgy A. Ermolaev, Andrey A. Vyshnevyy, Mikhail S. Mironov, Sergey M. Novikov, Aleksey V. Arsenin, Valentyn S. Volkov

**Affiliations:** 1 Center for Photonics and 2D Materials, Moscow Institute of Physics and Technology, 9 Institutsky Lane, Dolgoprudny 141700, Russia; dmitrii.yakubovskii@phystech.edu (D.I.Y.); grudinin.dv@phystech.edu (D.V.G.); georgiy.ermolayev@phystech.edu (G.A.E.); andrey.vyshnevyy@phystech.edu (A.A.V.); mironov.ms@phystech.edu (M.S.M.); novikov.s@mipt.ru (S.M.N.); arsenin.av@mipt.ru (A.V.A.); 2 Laboratory of Advanced Functional Materials, Yerevan State University, Yerevan 0025, Armenia

**Keywords:** ultrathin metal films, s-SNOM, MoS_2_, surface plasmon polaritons, dielectric constants

## Abstract

Ultrathin metal films are an essential platform for two-dimensional (2D) material compatible and flexible optoelectronics. Characterization of thin and ultrathin film-based devices requires a thorough consideration of the crystalline structure and local optical and electrical properties of the metal-2D material interface since they could be dramatically different from the bulk material. Recently, it was demonstrated that the growth of gold on the chemical vapor deposited monolayer MoS_2_ leads to a continuous metal film that preserves plasmonic optical response and conductivity even at thicknesses below 10 nm. Here, we examined the optical response and morphology of ultrathin gold films deposited on exfoliated MoS_2_ crystal flakes on the SiO_2_/Si substrate via scattering-type scanning near-field optical microscopy (s-SNOM). We demonstrate a direct relationship between the ability of thin film to support guided surface plasmon polaritons (SPP) and the s-SNOM signal intensity with a very high spatial resolution. Using this relationship, we observed the evolution of the structure of gold films grown on SiO_2_ and MoS_2_ with an increase in thickness. The continuous morphology and superior ability with respect to supporting SPPs of the ultrathin (≤10 nm) gold on MoS_2_ is further confirmed with scanning electron microscopy and direct observation of SPP fringes via s-SNOM. Our results establish s-SNOM as a tool for testing plasmonic films and motivate further theoretical research on the impact of the interplay between the guided modes and the local optical properties on the s-SNOM signal.

## 1. Introduction

Ultrathin metal films are essential elements of novel optoelectronic, photonic, and plasmonic devices [1,2,3]. Thanks to their transparency, conductivity, and mechanical stability, gold, silver, or copper ultrathin films of thicknesses below 10 nm are a promising platform for developing flexible transparent electrodes in various optoelectronic applications [4,5]. For example, plasmonic waveguides, hyperbolic metamaterials, and transparent electrodes made from ultrathin films should possess smooth and continuous structural morphology to have a superior optical response and electronic properties [6,7,8]. To realize ultrathin film growth, the conventional three-dimensional (3D) mechanism in the Volmer-Weber regime [9] should be avoided by using special deposition conditions [10] or inserting additional wetting layers [11]. One of the competing methods to obtain continuous two-dimensional (2D) metal growth on a substrate is employing atomically thin two-dimensional (2D) underlayers, such as monolayer or few-layer graphene or transition metal dichalcogenide (TMD), which favors continuous metal growth kinetics [12,13,14]. Among them, molybdenum disulfide (MoS_2_) has received large attention due to its tunable bandgap, high refractive index, and giant optical anisotropy resulting in a wide range of applications from field-effect transistors to solar cells and photodetectors [15,16,17,18,19]. In this regard, investigation of Au/MoS_2_ or other TMD interfaces is of great practical interest.

Recently, 2D MoS_2_ layers demonstrated impressive results of gold ultrathin film growth. Due to the surface wetting effect and the adhesion of gold to MoS_2_, high nucleation density and unusual kinetics of Au adatoms on MoS_2_ surface lead to the growth of continuous and ultrasmooth clusters and highly-percolated structures [13,20,21,22]. In recent papers, a detailed investigation of metal growth on MoS_2_ revealed the formation of atomically flat Au clusters, nanotriangles, and nanoparticles depending on the deposition technique, metal thickness, and temperature [20,21,23,24,25,26,27]. For example, the presence of MoS_2_ could enhance the performance of ultrathin noble metal electrodes and their optical response [22,25,28,29,30]. At the same time, the decoration of 2D MoS_2_ layers with nanoparticles helps to control and improve the characteristics of transistors, photodetectors, and Raman sensors [23,31,32]. However, despite its great importance, there is still no detailed study of continuous ultrathin gold films grown on exfoliated MoS_2_.

Investigation of the optical response of continuous gold films grown on small (~1 μm) flakes of MoS_2_ and nanoscale structures via far-field optical techniques, such as spectroscopic ellipsometry and reflectometry, is challenging. Nevertheless, one can study the optical properties on the nanometer scale via scanning optical probe methods. Among them, s-SNOM is a powerful technique for probing the optical response in material with nanoscale resolution [33]. In particular, s-SNOM was successfully utilized for the characterization of thin films [34], nanoparticles [35,36], optoelectronic devices [37], surface enhanced Raman spectroscopy biosensors [38,39] and layered van der Waals materials, such as graphene and TMDs [40,41,42]. Furthermore, in plasmonics and photonics, s-SNOM is an effective tool for the excitation and detection of surface plasmon and phonon polaritons in layered structures [43]. The detected s-SNOM signal strongly depends on the optical response of the tip-sample interaction area, which governs the scattering efficiency of light by the tip [44,45]. As a result, the light scattering efficiency by the tip in the near-field is stronger on metal with a higher modulus of the real part of permittivity [46,47]. In practice, this results in a high contrast in s-SNOM amplitude and phase between the metal and dielectric areas of the sample, which are distinguishable with nanoscale spatial resolution [45,46]. This fact allows to perform mapping of the local homogeneity of dielectric permittivity of the material, analyze the quality of the sample morphology, and identify sub-diffraction features on the surface [45,48,49]. Our previous work demonstrated that, for ultrathin gold films grown on different 2D materials, the s-SNOM near-field intensity correlates well with their far-field dielectric response [50].

This study demonstrates the transformation of gold film morphology during growth on exfoliated MoS_2_ flakes. Furthermore, morphological study with scanning electron and atomic-force microscopies was extended with s-SNOM near-field studies, which allowed to demonstrate of a qualitative improvement of plasmonic properties and structural evolution of metallic films on MoS_2_, which is crucial for next-generation ultrathin metal optoelectronics.

## 2. Materials and Methods

For the deposition of gold films, MoS_2_ 2D-crystal flakes were prepared via mechanical exfoliation on SiO_2_/Si wafer using scotch tape and bulk MoS_2_ crystal (2D semiconductors). To remove scotch residuals, the exfoliated flakes with substrates were carefully cleaned in acetone prior to deposition. Then, gold films were deposited via the electron-beam evaporation method in a high vacuum (~10^−6^ Torr) chamber setup (Nano-Master NEE-4000, Austin, TX, USA) at room temperature onto the prepared substrate of MoS_2_ flakes/SiO_2_. The mass-equivalent thicknesses of gold in the range of 4–10 nm and the deposition rate of ~1 A/s were measured in-situ via a calibrated quartz-crystal sensor.

The crystal quality of MoS_2_ flakes and the number of atomic layers in them were assessed by confocal Raman microscope Horiba LabRAM HR Evolution (Horiba Ltd., Kyoto, Japan). All measurements were carried out with laser excitation wavelengths of 632.8 nm with an 1800 lines/mm diffraction grating and a ×100 (N.A. = 0.90) objective. The spot diameter was ~0.5 µm.

The structural morphology of the deposited Au films was visualized via scanning electron microscopy (SEM, JSM7001F, JEOL, Tokyo, Japan) working in the secondary electron imaging mode with a Schottky emitter, an acceleration voltage of 30 kV and a working distance of ~6 mm. To characterize the surface roughness and homogeneity of films, we performed atomic force microscopy (AFM, Ntegra II, NT-MDT Spectrum Instruments, Moscow, Russia) working in the peak-force mode with a tip of radius <10 nm and a resonant cantilever frequency of ~140 kHz (NT-MDT, ETALON, HA_NC).

Optical near-field characterization of Au films grown on MoS_2_ flakes was performed using a scattering-type scanning near-field optical microscope (s-SNOM, neaSNOM, Neaspec GmbH, Haar, Germany) working in the reflection mode [46,47]. In this setup, we used a standard AFM tip coated with platinum, working in the tapping mode with a resonance frequency of *Ω* ≈ 280 kHz. The s-SNOM tip was illuminated via a focused linearly polarized laser beam (*λ* = 1550 nm) with an angle to the sample surface of about 50°. Next, the near-field was scattered by the tip interacting with the surface and redirected by an upper parabolic mirror to a highly sensitive photodetector. In addition, a pseudo-heterodyne interferometric scheme and demodulation of the detected scattered signal at higher harmonics (4 Ω) were used for acquiring a near-field signal filtered from the background contribution.

## 3. Results and Discussion

**Figure 1:**
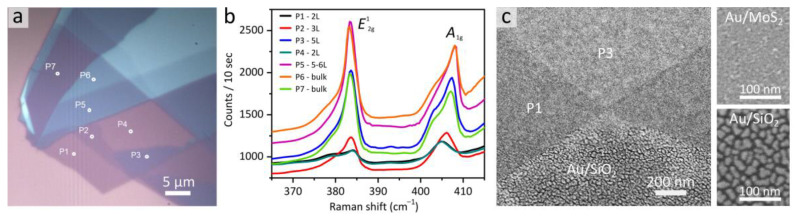
Optical microscopy (**a**) and Raman characterization (**b**) of a typical transferred few-layer MoS_2_ flake on the SiO_2_/Si substrate. Various colors in the optical image are attributed to different thicknesses of MoS_2_, i.e., 2 L, 3 L, and thicker MoS_2_ layers. The Raman spectra with a 632.8 nm excitation laser were collected from a spot size of about 1 µm at each measurement point at n-layered MoS_2_. (**c**) SEM image of the area across the Au/SiO_2_-Au/MoS_2_ flake surface boundary after deposition of a 4-nm-thick Au film, small SEM images represent the surface morphology of gold on MoS_2_ and SiO_2_.

First, few-layered MoS_2_ flakes were located on the SiO_2_ (285 nm)/Si substrate by their color contrast using an optical microscope. A typical optical image of the prepared flake with terraces consisting of a few MoS_2_ layers is shown in Figure 1a. The crystal quality and the number of layers were determined via Raman spectroscopy of the different areas marked with white dots in Figure 1a. The obtained Raman spectra exhibited typical MoS_2_ peaks *E*^1^_2g_ (~384 cm^−1^) and *A*_1g_ (~407 cm^−1^). The observed broadening and shifts of the distance between *E*^1^_2g_ and *A*_1g_ Raman modes in the spectra in Figure 1b indicated an increase in MoS_2_ thickness, consistent with the previously reported results [51].

**Figure A1:**
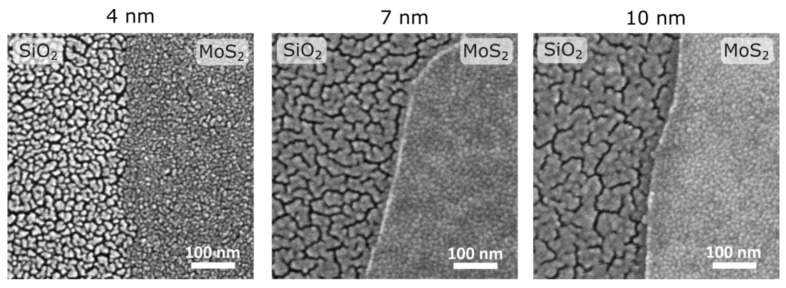
The comparison SEM images for gold films grown on SiO_2_ and MoS_2_ for 4 nm, 7 nm, and 10 nm film thickness.

To demonstrate the drastic difference in the morphology of Au film on MoS_2_ and SiO_2_ underlayers, a SEM image of the boundary between Au/MoS_2_ and Au/SiO_2_ was recorded (see Figure 1c and Figure A1). Clearly, ultrathin gold films on MoS_2_ are continuous and without voids, while ultrathin gold films on SiO_2_ are composed of particles separated by voids. A similar difference in the morphology of gold on MoS_2_ and SiO_2_ was previously observed for gold films on MoS_2_ monolayers grown by chemical vapor deposition (CVD) [22]. As can be seen, microscopy images show no visible variation in the morphology of Au grown on the different terraces of MoS_2_.

**Figure 2:**
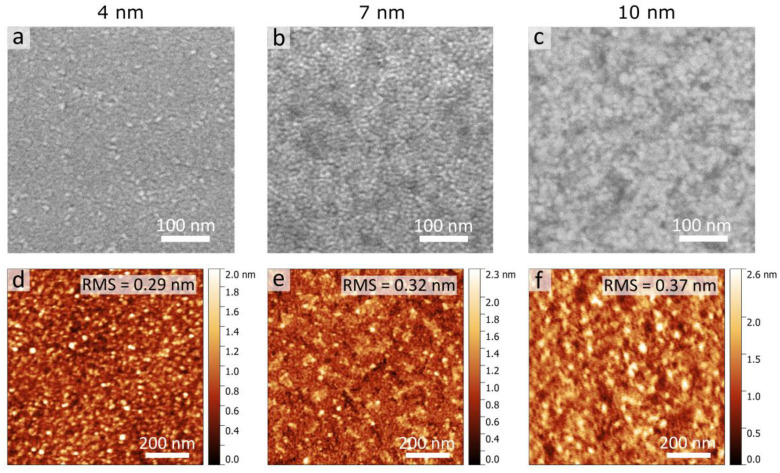
SEM surface images (**a**–**c**) and AFM topography scans (**d**–**f**) of Au films of varying thickness on few-layer MoS_2_ flakes. Values of RMS surface roughnesses of the Au films are indicated within AFM images. The AFM scan area is 1 × 1 μm^2^.

After the metal deposition, the surface morphology of the Au films grown on few-layer MoS_2_ was further examined via SEM and AFM. As seen in Figure 2, both the SEM (a–c) and AFM (d–f) images indicate that 4–10-nm-thick Au films on few-layer MoS_2_ fully cover the substrate. The polycrystalline structure of Au films was confirmed via SEM images as in Figure 2a–c. The full area metal coverage is due to a low percolation threshold and high density of nucleation sites at the initial stage of the film growth on MoS_2_. AFM surface analysis demonstrated in Figure 2d–f revealed an atomically smooth surface with a root-mean-square (RMS) roughness below 0.37 nm for all considered thicknesses, which additionally confirms full substrate coverage by the gold film. At the same time, surface roughness slightly increases with the increase in gold layer thickness, which is typical for continuous polycrystalline Au films [52,53].

**Figure 3:**
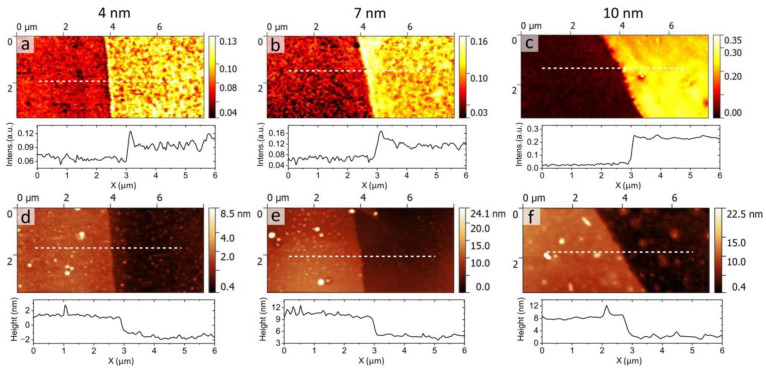
The measured s-SNOM amplitude scans (**a**–**c**) across the boundary between Au/SiO_2_ and Au/MoS_2_ areas for three different thicknesses with the intensity of signal cross-sections, related to the dotted white line, in arbitrary units below and corresponding scans of AFM topography (**d**–**f**) with height profiles below.

To probe the local optical response of gold films, we performed s-SNOM microscopy. For the measurements, we chose areas that include the boundary between Au/SiO_2_ and Au/MoS_2_ which allowed us to visualize the contrast between the optical properties of different gold films. Moreover, we focused on monolayer MoS_2_ underlayers rather than multilayer MoS_2_ to minimize the influence of MoS_2_ on the s-SNOM amplitude. The s-SNOM scans of the boundary between Au/MoS_2_ and Au/SiO_2_ for Au layer thicknesses of 4, 7 and 10 nm are demonstrated in Figure 3a–c. Here, the near-field amplitude showed a high contrast between Au films grown on the MoS_2_ and SiO_2_ surface for all thicknesses, with an s-SNOM amplitude at the area of Au/MoS_2_ being significantly higher than that for discontinuous film Au/SiO_2_. Furthermore, the contrast between Au/MoS_2_ and Au/SiO_2_ grows with the increase in the gold thickness, starting from about 50% for 4-nm-thick films (Figure 3a) to an order of magnitude for 10-nm-thick films (Figure 3c).

Higher quality of Au/MoS_2_ films on CVD MoS_2_ was previously established by the far-field measurements [22] and is qualitatively evident from Figure 1c. Nevertheless, for the additional confirmation we analyzed topography scans presented in Figure 3d–f. We clearly see the existence of a 3–5-nm-high step on the boundary between Au/SiO_2_ and Au/MoS_2_, in agreement with the previous AFM measurements [20]. Such a difference in thickness can be explained by the higher porosity of Au/SiO_2_ films which makes the total volume of Au/SiO_2_ film higher than that of Au/MoS_2_ film despite the same quantity of metal per unit area being deposited on each substrate. Interestingly, there are correlations between the topography and amplitude maps; for example, some bright spots on the left side of Figure 3d,e turn into dark spots in Figure 3a,b. Moreover, the fluctuation of the near-field amplitude of Au/MoS_2_ films grows from 16.8⋅10^−3^ to 21.5⋅10^−3^ as their thickness increases. Similar behavior is observed for topography scans that show the increase in the surface roughness of Au/MoS_2_ from 0.29 to 0.37 nm (Figure 2d–f). These results suggest that the near-field amplitude represents the local optical properties and morphology of gold films, in the spirit of the seminal work [45].

**Figure 4:**
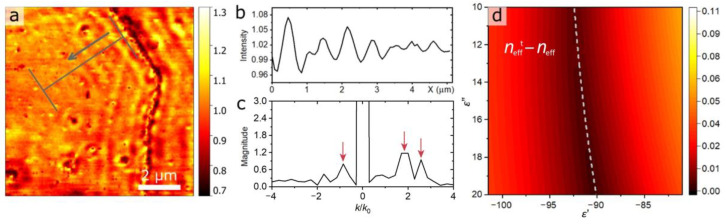
(**a**) s-SNOM amplitude imaging of fringes observed on 10-nm-thick Au film on MoS_2_ near the line-crack edge. (**b**) Signal profile line scan of the fringes extracted from (**a**) and averaged over 2.5 μm (gray line in **a**). (**c**) FT near-field amplitude of the near-field signal along propagation in (**b**). (**d**) Results of *ε*′ and *ε*″ range determination by comparison of the calculated effective mode index (*n*_eff_^t^) with the experimentally determined one.

Apart from local optical response distribution, s-SNOM could give additional information about ultrathin film properties as a whole. Indeed, the s-SNOM signal on the surface of the metal depends not only on the scattering properties of the SNOM tip and a sample area immediately underneath it but also on the guided SPP waves that can be excited by the tip and propagate up to dozens of micrometers along the film. The excitation of guided SPP is detectable via near-field fringes typically visible near the line cracks and other defects. Interestingly, despite the straight-line boundary between Au/MoS_2_ and Au/SiO_2_, no interference fringes are visible in Figure 3a,b. By contrast, a large 10 × 10 μm^2^ area scan of 10-nm-thick Au/MoS_2_ film reveals a line defect and a fringe pattern around it (see Figure 4a). These fringes clearly indicate the capability of the Au/MoS_2_ film to guide SPP waves. At the same time, the absence of fringes in Figure 3a–c can be related to the porous nature of Au/SiO_2_ film. Below the percolation threshold, the propagation of SPP waves can occur due to the dipole-dipole coupling between adjacent metallic islands, while above the percolation threshold the SPP wave is scattered by the disordered voids in the metal film. As a result, in both cases the phase coherence of SPP waves is rapidly lost, which, combined with the local fluctuations of optical properties, produces noisy amplitude maps in Figure 3a,b.

Next, using the capabilities of our s-SNOM setup to measure both the amplitude and phase of the near-field signal, we analyze them to retrieve an effective-mode index of propagation of surface plasmon modes. We carried out a one-dimensional complex Fourier transformation (FT) of the averaged s-SNOM signal along the SPP propagation, as illustrated in Figure 4a by the arrow, which allowed us to extract effective mode indices. As indicated in Figure 4c (the red arrows), the Fourier spectrum has three peaks *k*/*k*_0_ ≈ 1.87, 2.56 and −0.85; due to the geometry of the reflection mode s-SNOM setup, the “real” values *k*/*k*_0_ of the modes are shifted from the *k*/*k*_0_ observable in the experiment [54]. The value of the shift can be expressed as:

*β*_0_ = *k*_R_ + *k*_0_·cos *α*·sin *β*    *β*_0_/*k*_0_ = *k*_R_/*k*_0_ + cos *α*·sin *β*, (1)

*β*_0_ = *k*_L_ − *k*_0_·cos *α·*sin *β*     *β*_0_*/k*_0_
*= k*_L_*/k*_0_ – cos *α·*sin *β*, (2)

where *β_0_* corresponds to the observable mode, *k*_R_/*k*_0_ stays for the “real” effective mode index for the modes on the right side of the Fourier transform, *k*_L_/*k*_0_-for the left side, *α* is the angle between the wave vector of the incident light and its projection on the sample surface and *β* is the angle between the projection of the wave vector and the crack from which the light scatters. In our case, the shift is 0.86 due to the geometry of the experimental setup. According to Equations (1) and (2), we observe two propagating modes in the Fourier spectrum: one with effective-mode index 1.71 (*β*_0_/*k*_0_ ≈ 2.56 and *β*_0_/*k*_0_ ≈ −0.85) and the other with 1.02 (*β*_0_/*k*_0_ ≈ 1.87). The latter propagates along the air/Au interface (*k*/*k*_0_ ≈ 1.02) while the former along the interface between Au and the substrate (*k*/*k*_0_ ≈ 1.71).

In addition, we verified experimental effective-mode indexes by finding the film dielectric constants *ε*′ and *ε*″ via comparison of experimentally determined *n*_eff_ with theoretically calculated *n*_eff_^t^. For this, we performed full-wave electromagnetic simulations using COMSOL Multiphysics to obtain *n*_eff_^t^ selecting different values of dielectric functions *ε*′ and *ε*″ of 10-nm-thick gold film at a single wavelength of *λ* = 1550 nm. Results of the calculated difference *n*_eff_^t^ − *n*_eff_ are presented in Figure 4d. Here, the darker the color of the map, the closer the modes that are obtained for the *ε*′ and *ε*″ data are to the effective mode indices that are observed in the experiment. It is seen that the effective mode index mainly depends on *ε*′. At the same time, *ε*″ is responsible for propagation losses, which are difficult to determine from s-SNOM map, thus can be varied within a reasonable range. From Figure 4d, the ranges for the dielectric function of gold are |*ε*′| = 90–92.5, *ε*″ = 10–20 (darkest areas in Figure 4d indicated by the white dotted line) which is in agreement with the formerly measured dielectric constants *ε*′ = −95.3 and *ε*″ = 14.5 at *λ* = 1550 nm of 9-nm-thick gold film grown on MoS_2_-CVD monolayer [22]. To sum up, the high quality of Au/MoS_2_ films was demonstrated by s-SNOM image contrast measurements in Figure 3c, SEM in Figure 2, and by the observation of SPP fringes in Figure 4a. Also, we determined the effective index of SPPs propagating along the 10-nm-thick gold film, and, based on it, extracted the effective values of *ε*′ and *ε*″ of the gold film, which agree with the previous measurements.

## 4. Conclusions

We studied morphology and local optical response of ultrathin gold films deposited on SiO_2_ and 2D MoS_2_ crystal layers. The results demonstrate the significant contrast in s-SNOM amplitude between Au films on MoS_2_ and SiO_2_ for all measured thicknesses. As was observed, strong contrast in the s-SNOM signal is related to the different surface morphology and continuity of Au films deposited on SiO_2_ and MoS_2_ surfaces, which correlates well with the previously reported far-field and near-field investigations. Furthermore, we detected SPP wave propagation at the telecom wavelength range via s-SNOM imaging of near-field fringes from edge defect in 10-nm-thick Au film on MoS_2_, and theoretically calculated possible dielectric permittivities of gold. The observation of SPP propagation additionally confirms the high quality of the obtained ultrathin films. As a result, by obtaining nanoscale optical images, we demonstrated s-SNOM to be an effective method for analysis of the structural quality and optical response, which can give information about the propagation of surface waves in ultrathin metal films. We believe that our research will encourage the future use and deep theoretical study of s-SNOM for advanced nanoscale optical characterization of the optical and structural performance of metal-2D material interfaces.

## Data Availability

The data presented in this study are available on request from the corresponding author.
